# Transcriptome Sequencing of the Striped Cucumber Beetle, *Acalymma vittatum* (F.), Reveals Numerous Sex-Specific Transcripts and Xenobiotic Detoxification Genes

**DOI:** 10.3390/biotech9040021

**Published:** 2020-10-27

**Authors:** Michael E. Sparks, David R. Nelson, Ariela I. Haber, Donald C. Weber, Robert L. Harrison

**Affiliations:** 1 Invasive Insect Biocontrol and Behavior Laboratory, USDA-ARS, Beltsville, MD 20705, USA; michael.sparks2@usda.gov (M.E.S.); ariela.haber@usda.gov (A.I.H.); don.weber@usda.gov (D.C.W.); 2 Department of Microbiology, Immunology and Biochemistry, University of Tennessee Health Science Center, Memphis, TN 38163, USA; drnelson1@gmail.com

**Keywords:** striped cucumber beetle, sex-specific transcripts, xenobiotic detoxification, resistome, glutathione S-transferase, carboxylesterase, cytochrome P450, transcriptomics

## Abstract

*Acalymma vittatum* (F.), the striped cucumber beetle, is an important pest of cucurbit crops in the contintental United States, damaging plants through both direct feeding and vectoring of a bacterial wilt pathogen. Besides providing basic biological knowledge, biosequence data for *A. vittatum* would be useful towards the development of molecular biopesticides to complement existing population control methods. However, no such datasets currently exist. In this study, three biological replicates apiece of male and female adult insects were sequenced and assembled into a set of 630,139 transcripts (of which 232,899 exhibited hits to one or more sequences in NCBI NR). Quantitative analyses identified 2898 genes differentially expressed across the male–female divide, and qualitative analyses characterized the insect’s resistome, comprising the glutathione S-transferase, carboxylesterase, and cytochrome P450 monooxygenase families of xenobiotic detoxification genes. In summary, these data provide useful insights into genes associated with sex differentiation and this beetle’s innate genetic capacity to develop resistance to synthetic pesticides; furthermore, these genes may serve as useful targets for potential use in molecular-based biocontrol technologies.

## 1. Introduction

**Figure 1:**
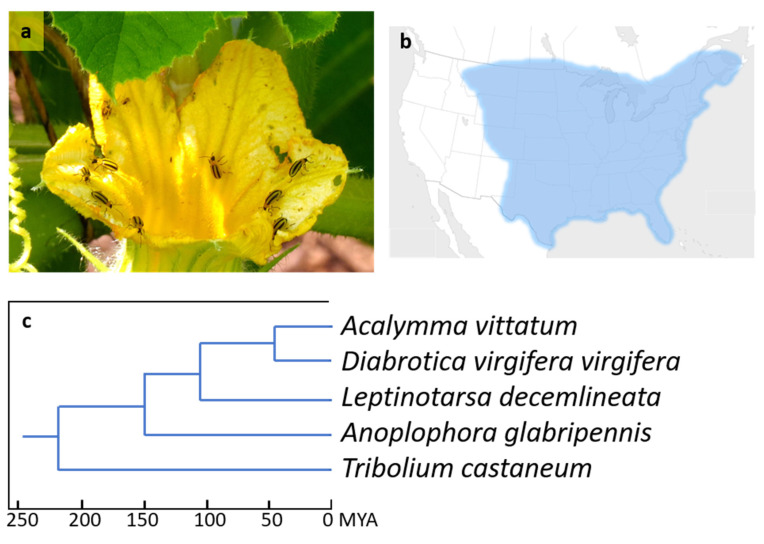
Feeding example, host range, and related species of *Acalymma vittatum*, the striped cucumber beetle. (**a**) Adult *A. vittatum* feeding on cucurbit flower (photo by D. Gordon E. Robertson, Ottawa, Ontario); (**b**) geographic occurrence of *A. vittatum* (based on Figure 1 of [2]); (**c**) cladogram of Coleopteran species (based on Suppl. Figure 8 of [18], and Figure 2 and Table 3 of [19]).

The striped cucumber beetle, *Acalymma vittatum* (F.) (Coleoptera: Chrysomelidae), is a specialist herbivore of cucurbit crops, native to North America east of the Rocky Mountains. It is the primary pest of these crops in northeastern and midwestern USA and eastern Canada, mainly through damage to seedlings and young plants [1,2], as well as transmission of the virulent bacterial wilt pathogen *Erwinia tracheiphila* [3] (Figure 1). Adults feed primarily on above-ground parts of Cucurbitaceae and larvae primarily underground. There are one to three generations per year, with adults being the overwintering stage. Females lay a total of several hundred eggs near the base of host plants. Larvae have three instars lasting a total of 11 to 45 days [1,2]. Like many species in the tribe Luperini, including the related genus *Diabrotica*, *A. vittatum* are arrested by and stimulated to feed on cucurbitacins, the bitter compounds present in their host plants. Adults are notorious for their ability to rapidly colonize newly-planted squash, pumpkin, and cucumber fields. These aggregations form in response to the aggregation pheromone, vittatalactone, produced by feeding males [4,5].

Management of striped cucumber beetles is primarily with broad-spectrum insecticides such as pyrethroids and neonicotinoids. However, chemical control is not always effective because of the mobility of adults and the cryptic feeding of the larvae, and it poses a distinct risk to the pollinators necessary for production of the harvested fruits [1]. Other management tactics [2,6,7,8] include transplanting instead of direct seeding, to reduce plant vulnerability in the field, physical protection with row covers up until flowering, trap cropping by planting border rows of highly attractive cultivars, intercropping, and mulching. The pilot production of vittatalactone offers the future possibility of monitoring, mass trapping, and/or attract-and-kill baiting using vittatalactone and cucurbitacins [9]. An attractive complementary control technique consists of the development of species-specific molecular biopesticides, specifically double-stranded RNA molecules that can be used to effect knockdowns of one or more targeted genes via RNAi pathways, manifesting in a pest phenotype with reduced fitness [10,11].

Prerequisite to the design of such molecular resources is a comprehensive understanding of the pest insect’s gene repertoire, achievable through high-throughput transcriptome and/or genome sequencing and analysis. Interrogations of insect gene space with a deliberate attempt to identify RNAi targets have been conducted in Lepidoptera [12], Hemiptera [13,14,15], and Hymenoptera [16], among others; the present work extends upon related efforts within the Coleoptera ([17]). Sex-specific genes offer a useful target for potentially disrupting host reproduction, whereas genes pertinent to xenobiotic detoxification (i.e., the “resistome”, comprising glutathione S-transferases, carboxylesterases, and cytochrome P450 monooxygenases) impede an insect’s capacity to tolerate traditional, chemical insecticides. This study identified 2898 differentially expressed *A. vittatum* genes and phylogenetically analyzed its resistome in the context of four related Coleopteran species (Figure 1).

## 2. Materials and Methods

Adult beetles were collected from a squash field at the Beltsville Agricultural Research Center in Beltsville, MD, USA (39°01′01″; W76°56′31″) on 7 and 11 August 2017. Beetles were immediately frozen in liquid nitrogen and stored at −80 °C until RNA was extracted. Specimens were sexed, and three biological replicates—consisting of five pooled individuals, in accord with previous practice using similarly sized insects [13,14]—were prepared for each sex. Pools of five beetles were homogenized separately in 2.0 mL tubes containing Lysing Matrix A (MP Biomedicals, Solon, OH, USA) and Lysis/Binding Solution from the mirVana™ miRNA Isolation Kit (ThermoFisher Scientific, Waltham, MA, USA) using a FastPrep-24™ Tissue and Cell Homogenizer (MP Biomedicals, Solon, OH, USA) set at 4.0 m/s and run for 40 s. Insoluble material was pelleted by centrifugation, and total RNA was recovered from the supernatants with the mirVana™ miRNA Isolation Kit. RNA samples were submitted for PE150 sequencing on an Illumina HiSeq instrument operated by the Georgia Genomics Facility (Athens, GA, USA). Table 1 presents sequencing volumes achieved after quality-based read trimming, performed by the sequencing vendor. Trimmed reads were pooled, normalized, and assembled using Trinity (version 2.6.6 [20]). Raw reads and the assembled transcriptome are available from NCBI’s SRA and TSA divisions, respectively, under BioProject accession number PRJNA493105.

Differentially expressed genes (DEGs) were identified using DESeq2 (version 1.18.1 [21]) in conjunction with the salmon read abundance quantification tool operating in its quasi-alignment mode [22]. The tximport package [23] was used to develop gene-level counts as a function of transcript-level data, and differential analysis was performed on this two-factor dataset using DESeq2’s *DESeq* function. Among genes salmon identified as having non-zero expression levels, a candidate was flagged as differentially expressed if it exhibited at least a two-fold difference in expression levels between males and females, and had an adjusted p-value of 0.05 or less (alpha = 0.05, lfcThreshold = log2(2), altHypothesis = “greaterAbs”). At the transcript level, expression was also estimated using the RSEM tool [24] in conjunction with the bowtie2 [25] aligner; transcript and gene abundances were conveyed using the transcripts per million measure (TPM, [26]).

Assembled mRNA pseudomolecules were compared with the 30 January 2019 version of the NCBI NR protein database using DIAMOND (version 0.9.22 [27]) in its BLASTx-like mode with default parameter settings. The top hit per query, if any, was recorded—if multiple best-scoring hits were encountered, an arbitrary selection was made. The subset of transcripts associated with DEGs was also compared to NR with BLASTx [28], to identify matches that may have been overlooked by the more-heuristic DIAMOND tool. Primers for potential use in synthesizing dsRNA probes were designed using PrimerPlex version 2.62 (PREMIER Biosoft, Palo Alto, CA, USA), both in cross-homology intolerant and tolerant modes.

The longest open reading frame was identified for each *A. vittatum* transcript and translated in silico using transeq from the EMBOSS package [29]. For the glutathione S-transferase (GST) and carboxylesterase (COE) gene families, representative sequences were obtained from four comparator taxa, on the basis of their respective genome annotations: *Anoplophora glabripennis* (Asian longhorned beetle, [30]; annotations obtained from NCBI as GCF_000390285.2_Agla_2.0_protein.faa); *Leptinotarsa decemlineata* (Colorado potato beetle, [31]; GCF_000500325.1_Ldec_2.0_protein.faa); *Tribolium castaneum* (red flour beetle, [32]; GCF_000002335.3_Tcas5.2_protein.faa); and *Diabrotica virgifera virgifera* (western corn rootworm, unpublished data; GCF_003013835.1_Dvir_v2.0_protein.faa.gz). Comparisons with inferred *A. vittatum* protein sequences were performed using BLASTp, and the full complement of proteins for the gene family was multiply aligned using MUSCLE [33]. Phylogenies were constructed with RAxML [34] using a JTT model of protein evolution for branch length estimation [35] and 100 bootstrap replicates for node support. Phylograms were visually rendered using FigTree [36]. Annotations of gene family members were based on both a sequence’s placement in the overall phylogenetic tree and its best BLASTp hits in the overall NCBI NR database. Annotated phylograms were exported from FigTree, from which newick-tools [37] was used to distill cladograms containing only *A. vittatum* leaf nodes.

For the Cytochrome P450 monooxygenase (CYP) family, the raw transcriptome assembly (containing 630,139 sequences) was formatted for NCBI standalone BLAST. The file was batch tBLASTn searched using 58 insect CYP sequences covering all beetle CYP families. The top 300 hits were retained in each search. Duplicates were filtered out, leaving 552 unique accessions, which were retrieved from the subject database using the fastacmd utility. These nucleotide sequences were translated on the Virtual Ribosome [38]. A few sequences, such as pseudogenes, were translated from the wrong strand and had to be manually corrected, but the majority were correctly translated. These peptide sequences were batch BLASTp searched against all named insect P450s in a confidential database. Sequences were sorted by best BLAST hit and assigned to CYP families and subfamilies. Twelve false positive hits and nine potential contaminants (one of possible plant and eight of possible nematode origin) were removed. The transcript set contained many isoforms of the same gene (such as DN64698_c3_g1, comprising variants i1 to i7); a best sequence was selected from these to remove duplicates. Two hundred sequences remained, from which 90 fragments, four partial genes, and five pseudogenes were removed to yield a 101-sequence set suitable for a phylogenetic tree (see “CYP_annotations.Avittatum.xlsx” in Supplementary Materials). For comparison purposes, CYPs from *T. castaneum*, *L. decemlineata*, *A. glabripennis*, and *D. v. virgifera* were included. These sequences were named based on BLAST results against all named insect CYPs. (*L. decemlineata* CYPs were named previously [39], but were updated by Genbank). Following the same methods described above for GSTs and COEs, a total of 521 CYP protein sequences were multiply aligned and used to build a tree.

## 3. Results

The Trinity-based global *A. vittatum* RNA-Seq assembly yielded 630,139 transcripts (391,295,690 bp total). Screening for putative microbial transcripts and other contaminants upon NCBI SRA submission reduced this to 621,936 transcripts composed of 384,972,765 bases. Comparison of transcripts to NCBI NR with DIAMOND resulted in a total of 54,233 unique protein identifiers listed as best matches for 232,899 distinct transcripts (i.e., ~37.0% of the unfiltered total).

### 3.1. Sex-Specific Gene Expression

Salmon/DESeq2-based, gene-level analyses among 290,005 genes with a non-zero read count indicated 1896 genes were more abundantly expressed in males and 1002 in females—to wit, approximately 1.0% of candidate loci were flagged as differentially expressed genes (DEGs). Among 6086 transcripts associated with DEGs up-regulated in males, 2717 had NR matches per DIAMOND (44.6%) and 2152 were matched per BLASTx (35.4%). Among 3424 transcripts associated with female-preferential DEGs, these amounts were 1719 (50.2%) and 1285 (37.5%), respectively. A comprehensive listing of differentially expressed genes and their associated transcripts, with TPM-based expression levels as well as any NR protein hits and transcript-specific PCR primers (if found), is presented in the Supplementary file, “SCB_DEGcontrasts_genesANDisoforms.xlsx”. Table 2 presents select male- and female-preferential DEGs.

### 3.2. Resistome Characterization

#### 3.2.1. Glutathione S-Transferases

**Figure 2:**
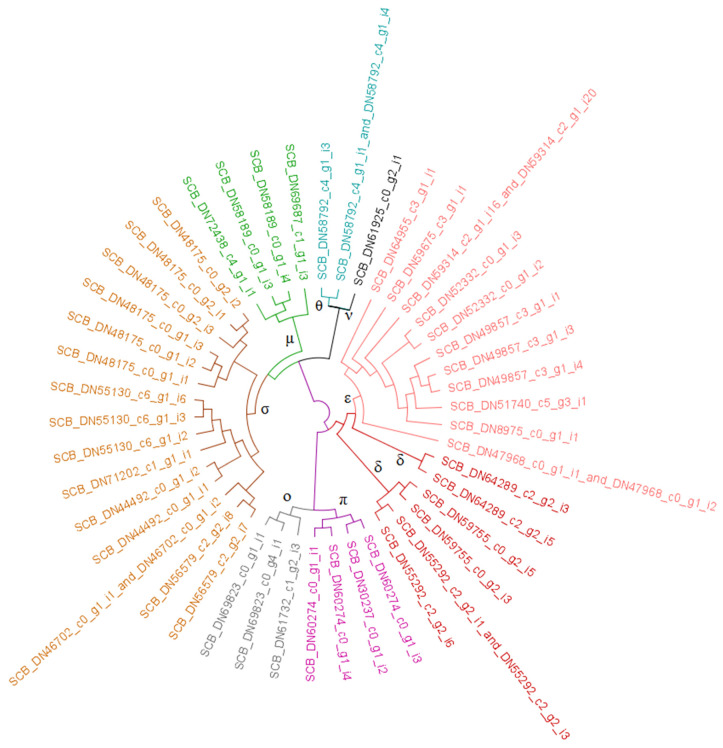
Glutathione S-transferase (GST) enzymes from *A. vittatum*. Cladogram of striped cucumber beetle GSTs distilled from the comprehensive, five-taxa phylogram presented in Figure S1. GST classes are connoted using branch and leaf coloring as specified below. To accommodate contexts in which color cannot be readily discerned, symbols are specified at branches to indicate that leaf nodes occurring in the subtree below are of a given class unless otherwise overridden by presence of an additional symbol. Blue (**θ**) ~ theta, green (**μ**) ~ microsomal, brown (**σ**) ~ sigma, grey (**ο**) ~ omega, purple (**π**) ~ prostaglandin E synthase, crimson-shaded red (**δ**) ~ delta, strawberry-shaded red (**ε**) ~ epsilon, and black (**ν**) ~ not classified.

Multiple subtypes of glutathione S-transferases (GSTs) exist, of which at least seven appear present in these transcriptome data. Figure 2 presents a cladogram of GST enzymes identified in *A. vittatum*, which has been distilled from the phylogram presented in Figure S1—in particular, Figure S1 comprehensively analyzes these genes in the context of five Coleopteran species. Four microsomal GST enzymes and seven prostaglandin E synthases were apparent in *A. vittatum*, which constitute all evident instances of the MAPEG (Membrane-associated Proteins in Eicosanoid and Glutathione Metabolism) protein family in this host [40]. Among the six cytosolic GST subclasses known in insects (Delta, Epsilon, Sigma, Theta, Omega, and Zeta [41,42]), all but the Zeta type were unambiguously observed. Eleven distinct Epsilon-type enzymes were observed in *A. vittatum*, which is roughly double the number of Delta class enzymes encountered (six distinct proteins). A total of 15 Sigma-class GSTs was observed, composing the second-most abundant grouping of GSTs in *A. vittatum* following the Delta/Epsilon composite group. Two distinct Theta-class enzymes were identified, which were situated in a clade of distinctive, single-copy GSTs that could not be conclusively classified but were present in all five beetle species surveyed (Figure 2 and Figure S1). Three Omega-class GSTs were observed in the *A. vittatum* transcriptome assembly.

#### 3.2.2. Carboxylesterases

**Figure 3:**
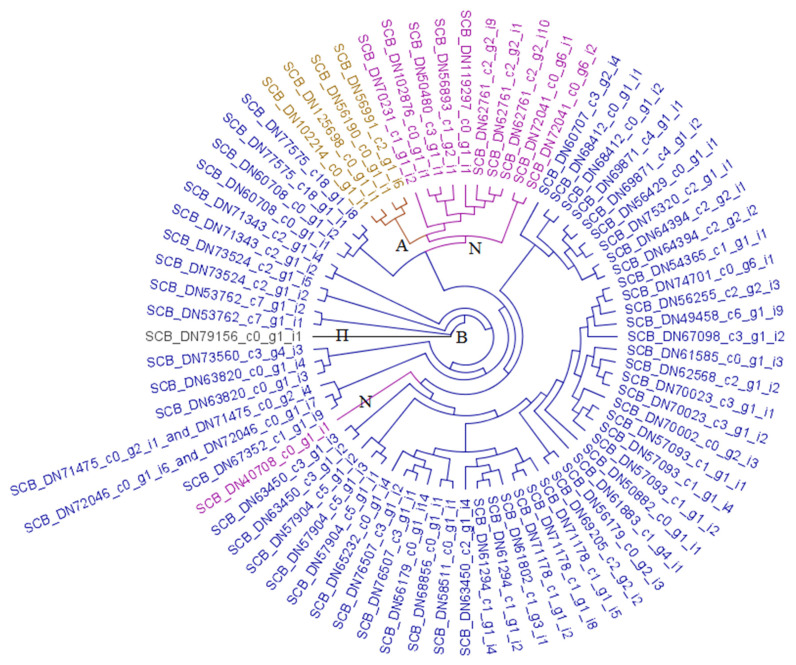
Carboxylesterase (COE) enzymes from *A. vittatum*. Cladogram of striped cucumber beetle COEs distilled from the comprehensive, five-taxa phylogram presented in Figure S2. COE classes are connoted using branch and leaf coloring as specified below. To accommodate contexts in which color cannot be readily discerned, symbols are specified at branches to indicate that leaf nodes occurring in the subtree below are of a given class unless otherwise overridden by presence of an additional symbol. Purple (**Ν**) ~ neuroligins, brown (**Α**) ~ acetylcholinesterases, royal blue (**Β**) ~ β-esterases, and black (**Π**) ~ palmitoleoyl COE NOTUM.

Figure 3 presents a cladogram of carboxylesterase (COE) enzymes identified in *A. vittatum*, distilled from the more comprehensive phylogram presented in Figure S2. Four acetylcholinesterases (ACEs) were detected in *A. vittatum*. A total of eleven *A. vittatum* neuroligins was observed, although these appear to consist of two quite distinct subtypes: ten of these occurred in a sister clade to the β-carboxylesterases (see below), while one instance, apparently a neuroligin-4 gene, was present in a subclade ensconced within the β-carboxylesterases clade proper. No *A. vittatum* neurotactin orthologs were detected in the transcriptome assembly. The β-carboxylesterases composed the most abundant COE subtype encountered in *A. vittatum*, presenting with 60 distinct enzyme types. A single-copy ortholog of the secreted palmitoleoyl COE NOTUM gene was present in the striped cucumber beetle.

#### 3.2.3. Cytochrome P450 Monooxygenases

**Figure 4:**
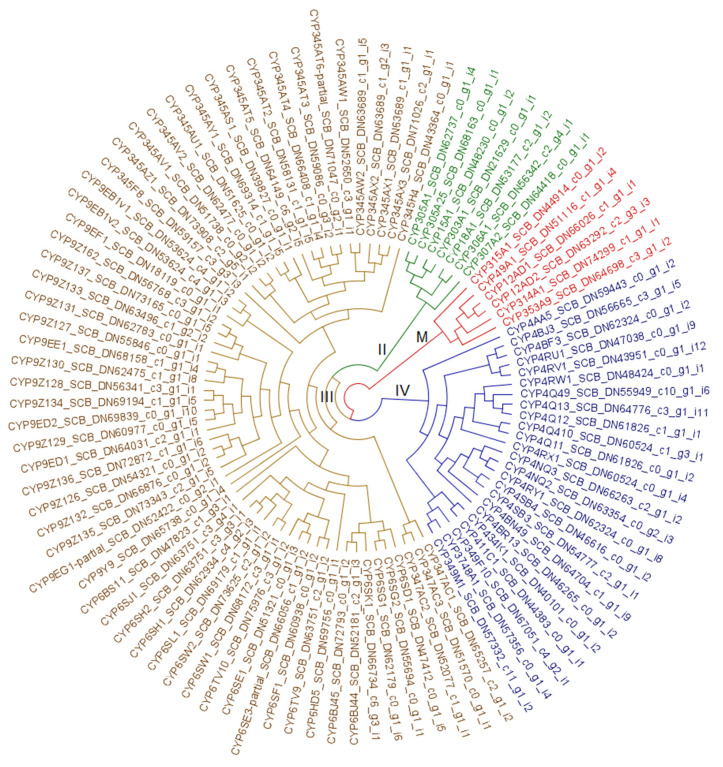
Cytochrome P450 monooxygenase (CYP) enzymes from *A. vittatum*. Cladogram of striped cucumber beetle CYPs distilled from the comprehensive, five-taxa phylogram presented in Figure S3. CYP clans are indicated using branch and leaf coloring as specified below. To accommodate contexts in which color cannot be readily discerned, symbols are specified at branches to indicate that leaf nodes occurring in the subtree below are of a given clan unless otherwise overridden by presence of an additional symbol. Green (**II**) ~ CYP2, brown (**III**) ~ CYP3, royal blue (**IV**) ~ CYP4, and red (**Μ**) ~ mito.

Figure 4 presents a cladogram of CYP enzymes identified in *A. vittatum*, distilled from the more comprehensive phylogram presented in Figure S3. The 98 striped cucumber beetle sequences used here for tree building belong to 21 families in the four canonical CYP clans: CYP2 (seven sequences), CYP3 (61 sequences), CYP4 (24 sequences), and mito (six sequences). Among these, the CYP3 clan is dominant, containing ~62% of *A. vittatum*’s P450s. The CYP9Z subfamily within the CYP3 clan contains 13 genes. The CYP345 family, also in the CYP3 clan, has 18 members distributed across ten subfamilies.

## 4. Discussion

Among the various motivations for this study was the search for *A. vittatum* genes involved in synthesis of its aggregation pheromone, vittatalactone [4,5]. Only males produce and release this pheromone, but it attracts both sexes. Vittatalactone is released when male beetles are feeding, and release occurs regardless of whether the beetles are feeding on pollen, cucumber containing cucurbitacin, or cucumber with no cucurbitacin. This suggests that the beetles synthesize the pheromone de novo, rather than from a specific plant precursor. Vittatalactone is structurally very similar to beta-lactone ring-containing enzyme inhibitors such as ebelactone A and F-244. These compounds are part of a large, structurally and biologically diverse group of natural products known as polyketides, whose synthesis is catalyzed by members of the polyketide synthase enzyme family from small (2- and 3-carbon) coenzyme A-linked precursors [43]. Several compounds in insects have been identified that appear to be products of polyketide biosynthesis on the basis of structural features and data from metabolic labeling experiments [44,45,46]. While numerous polyketide synthase-encoding genes have been identified from bacteria, fungi, and plants, few sequences encoding potential polyketide synthetic activities have been identified from insects [47]. Examples of insect polyketides that appear to be synthesized by symbiotic bacteria associated with insects have been documented [48,49,50]. Unfortunately, our analysis and comparison of male and female *A. vittatum* transcriptomes failed to produce convincing evidence for genes, either in the beetle itself or in a prospective symbiont, with a likely role in vittatalactone biosynthesis. This may reflect that the transcript encoding the vittatalactone biosynthetic enzyme was not being actively transcribed when RNA was collected from the insects, or that existing bioinformatics methods are not adequately sensitive to detect it in our dataset. More extensive sampling and/or sequencing of the host’s genome may help resolve this.

Multiple subtypes of glutathione S-transferases (GSTs) exist, of which at least seven appear present in these transcriptome data (Figure 2 and Figure S1). Four microsomal GST enzymes and seven prostaglandin E synthases were apparent in *A. vittatum*. Members of these two groups are classified as subtypes of the MAPEG protein family [40], which comprises membrane-bound GSTs. All remaining GST subclasses considered here are cytosolic and unbound [51]. At least six cytosolic GST subclasses are known in insects—Delta, Epsilon, Sigma, Theta, Omega, and Zeta [41,42,52]. Only the Delta and Epsilon subclasses are currently known to confer insecticide resistance [53]; Delta-type GSTs are observed across all the Insecta, whereas Epsilon-type genes have only been identified in the Holometabola (which contains the Coleoptera) [42]. Consistent with observations seen in *T. castaneum* [40], more distinct Epsilon-type enzymes (eleven in total) were observed in *A. vittatum* than were enzymes of the Delta class (six distinct proteins). Note, however, that bioinformatically distinguishing Delta GSTs from Epsilon on the basis of best BLASTp hits in NR references is potentially error-prone (as the exemplars themselves may be improperly annotated), and so the classifications proffered here are naturally tentative until more exhaustive biochemical characterizations have been conducted.

A total of 15 Sigma-class GSTs was observed, composing the second-most abundant grouping of GSTs in *A. vittatum* following the Delta/Epsilon composite group. These enzymes play a role in detoxifying toxic by-products of lipid peroxidation [54,55]. The functional role of Theta-class GSTs is not yet very well understood, although at least in *Bombyx mori*, these do not appear to play a role in oxidative stress response [56]. Two distinct Theta-class enzymes were identified; these were situated in a clade of distinctive GSTs that could not be unequivocally classified, but appeared to be single-copy in all five beetle species surveyed (Figure 2 and Figure S1). Although three Omega-class GSTs were observed in *A. vittatum*, the SCB_DN69823_c0_g4_i1 sequence is likely only partial (or misassembled): as it is, it lacks any cysteine residues, which are thought to be present in the active sites of all Omega-class GSTs [57]. Genome sequencing and spliced alignment of this encoding transcript onto its host gene’s chromosomal locus would clarify this. Although Zeta group enzymes have been observed as single-copy genes in other beetles [58], none were apparent in the *A. vittatum* transcriptome assembly. A multiple sequence alignment of the single-copy, unclassified GSTs depicted with black highlighting in Figure S1 with the three Zeta sequences of Mason et al. 2016 [58] indicated the former were positively not Zeta-class GSTs (results not shown).

Among the three principal subcategories of the carboxylesterase (COE) gene superfamily is a set comprising the catalytic ACE enzymes and various non-catalytic, neurodevelopment-associated genes, including neuroligins and neurotactins [59,60]. Point mutations of ACEs have been associated with insecticide resistance, in particular with carbamate and organophosphate compounds [61,62,63]. Four ACEs were detected in *A. vittatum*, each of which appear to have been transcribed from a unique genic locus (i.e., none of these are isoforms of the others per the Trinity assembler).

A total of eleven *A. vittatum* neuroligins were observed, although these appear to consist of two quite distinct subtypes: ten of these occurred in a sister clade to the β-carboxylesterases (see below), while one instance, apparently a neuroligin-4 gene, was present in a subclade ensconced within the β-carboxylesterases clade. Figure S2 suggests that within this neuroligin-4 group, the striped cucumber beetle and western corn rootworm contain single copies of these genes, whereas the Asian longhorned and Colorado potato beetles contain two paralogous copies, and *T. castaneum* has no copies. The placement of this group may be an artifact arising from the long branch lengths of the clade’s members (among the longest observed in the phylogeny). However, adjustment of tree building parameters failed to place it in a distinct region of the phylogram (results not shown), and inspection of top NCBI NR hits and associated alignments per BLASTp suggest that members within this group are indeed more similar to themselves than other sequences, and that they form a highly distinct group among neuroligins. Although one or more neurotactin genes were present in all four comparator taxa (Figure S2), no *A. vittatum* neurotactin orthologs were detected in the transcriptome assembly. As is often the case with RNA-Seq studies, this may result from transcriptional quiescence under the conditions in which source insects were processed, or failure to assemble associated reads into a complete, correct transcript. Genome sequencing would likely help to identify any *A. vittatum* neurotactin homologs, if they exist.

Another principal subgroup of the COE gene family consists of semiochemical and hormone processing genes, and includes β-carboxylesterases, the duplication (or overexpression) of which has been shown to associate with insecticide resistance through an elevated level of insecticide-sequestering enzymes [59,60]. By far, the β-carboxylesterases composed the most abundant COE subtype encountered in *A. vittatum*, presenting with 60 distinct enzyme types. A single-copy ortholog of the secreted palmitoleoyl COE NOTUM gene, which functions in at least the Wnt cell signaling pathway [64], was present in the striped cucumber beetle, as was also the case for the four other species considered in the combined phylogeny of Figure S2. These genes formed a clade situated within the overall phylogeny’s β-carboxylesterase subtree, although their shared ancestral branch was quite long, indicating substantive divergence with respect to the β-carboxylesterases proper.

The 98 striped cucumber beetle CYP sequences used here for tree building belong to 21 families in the four canonical CYP clans: CYP2 (seven sequences), CYP3 (61 sequences), CYP4 (24 sequences), and mito (six sequences). The CYP2 and mito clans are the smallest and contain genes reserved mostly for endogenous substrates like ecdysone intermediates and conserved pathways, including the Halloween genes [65]. The CYP4 clan is a little larger, while the CYP3 clan is dominant, containing ~62% of *A. vittatum*’s P450s. The CYP9Z subfamily in the CYP3 clan stands out with 13 genes. As transcriptomic rather than genomic data were analyzed here, it is not possible to know if CYP9Zs are organized in a tandem array, although this seems likely. The CYP345 family, also in the CYP3 clan, has 18 members, but they are in ten subfamilies, indicating sequence divergence and probably greater age than the CYP9Z bloom. The CYP4G family contributes the function of making a hydrocarbon coating to waterproof the exoskeleton by decarbonylation of fatty acids [66]. However, *A. vittatum* CYP4G sequences were not included in the tree: these exist as 35 fragments in the transcriptome that could not be assembled into complete sequences. The majority of genes in the CYP3 and CYP4 clans are probably devoted to metabolizing exogenous substrates to chemically detoxify harmful substances encountered in the environment.

This effort generated an extensive catalog of information pertaining to sex-specific gene expression in a non-model and agriculturally important species. In particular, the abundance of preferentially male- and female-expressed transcripts lacking evident homologs among NCBI NR exemplar sequences suggests the dataset generated here affords many opportunities to characterize novel, sex-specific genes through, for instance, reverse genetic screens implemented by RNAi. This work complements previous transcriptomic studies of various beetle species, including qualitative investigations into cantharidin biosynthetic pathways in the blister beetle, *Mylabris cichorii* [67]; lignocellulose degradation-related enzymes of the bamboo weevil, *Cyrtotrachelus buqueti* [68]; and odorant binding and other chemosensory proteins of the white-striped longhorn beetle, *Batocera horsfieldi* (Hope) [69]. It also complements quantitative studies into expression differences across tissue types and developmental stadia in *T. castaneum* [70], across sexes in *M. cichorii* adults [67], and across tissues and sexes in mated vs. sexually naïve adults of *Callosobruchus maculatus* seed beetles [71]. The topic of a future study will be to find counterpart genes among these prior studies to the potential *A. vittatum* RNAi targets identified here, and to compare them interspecifically along the axes of expression level differences and, more importantly, sequence similarity levels. In particular, tailoring highly species-specific dsRNAs in the context of real-world RNAi-mediated biocontrol applications is crucial to minimize risks of disrupting off-target, and potentially ecologically beneficial, species [72], and this will critically depend on surveying homologous sequences across a diverse range of related species.

## 5. Conclusions

Although a vittatalactone biosynthetic enzyme seemingly must be present in this insect, transcriptome sampling failed to identify it, suggesting that its encoding transcript may only be expressed under very specific circumstances (e.g., within a certain window of time after feeding) or at very low levels, and that genome sequencing may be a better approach to determine its sequence. Among a set of 2898 genes differentially expressed across the male-female divide, only 1525 (i.e., ~52.6%) of these had one or more isoforms with a convincing match in NCBI NR per the DIAMOND aligner (1238 (~42.7%) per BLASTx); this suggests the presence of many novel sex-specific genes possessing unknown function. The extensive repertoire of Delta/Epsilon and Sigma class GSTs in the striped cucumber beetle underscores an innate potential for developing insecticide resistance. The presence of four acetylcholinesterases and at least 60 distinct β-carboxylesterases, as well as an extensive set of CYP4 and CYP3 P450 monooxygenases, also bespeaks of this nuisance insect’s capacity to acquire resistance to synthetic chemical pesticides, indicating that non-insecticide approaches are necessary for long-term control of this pest.

## Figures and Tables

**Table 1:** Sample-specific, post-quality trimmed sequencing volumes, and amounts used for global transcriptomic assembly.

	Male (♂)			Female (♀)			Pooled and Normalized
	Biorep A	Biorep B	Biorep C	Biorep A	Biorep B	Biorep C	
read pairs	87,090,530	104,200,730	104,805,615	97,979,570	83,184,389	79,066,591	32,489,733
R1 bases	348,362,120	416,802,920	419,222,460	391,918,280	332,737,556	316,266,364	4,887,723,739
R2 bases	348,362,120	416,802,920	419,222,460	391,918,280	332,737,556	316,266,364	4,896,385,761

**Table 2:** Ten male- and ten female-preferential differentially expressed genes (DEGs). Positive and negative fold change (fold ∆) values indicate male- and female-preferential expression, respectively. Records within these two groups are sorted in ascending order with respect to adjusted *P*-value (P-adj). Biological replicate-specific expression values, conveyed in transcripts per million (TPM), are presented for both sexes (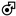 ~ male, 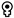 ~ female). Suggested gene function, as inferred on the basis of hits identified in NCBI NR, is also presented (NR Hits/ Inferred Function).

			TPM			TPM			
Gene ID	Fold ∆	P-Adj	♂ A	♂ B	♂ C	♀ A	♀ B	♀ C	NR Hits/ Inferred Function
DN48906_c127_g1	1.39 × 10^1^	4.86 × 10^−69^	2342.39	3149.43	2719.37	0.25	0.38	0.11	*none*
DN48785_c0_g1	1.37 × 10^1^	3.94 × 10^−66^	3550.32	4564.73	4809.53	0.40	0.55	0.43	*none*
DN52678_c6_g1	1.39 × 10^1^	3.65 × 10^−53^	1201.27	1448.84	1526.59	0.14	0.12	0.19	antichymotrypsin-2; alaserpin
DN47640_c7_g1	1.36 × 10^1^	1.85 × 10^−51^	1503.71	1670.18	1706.32	0.11	0.28	0.11	*none*
DN49285_c6_g1	1.36 × 10^1^	4.15 × 10^−49^	1353.96	1585.53	1467.49	0.07	0.05	0.19	uncharacterized protein
DN52414_c3_g1	1.23 × 10^1^	1.23 × 10^−47^	3039.40	3952.92	4415.55	0.74	0.86	0.63	*none*
DN69908_c0_g1	1.00 × 10^1^	9.96 × 10^−25^	9.97	7.91	5.41	0.02	0.00	0.01	ELKS/Rab6-interacting/CAST; kinectin
DN54481_c1_g3	9.47 × 10^0^	1.65 × 10^−24^	20.37	24.23	22.57	0.04	0.03	0.03	IQ domain-containing protein D
DN67865_c1_g1	1.19 × 10^1^	9.71 × 10^−24^	1174.38	1563.92	1652.00	0.08	0.28	0.35	cysteine rich trypsin inhibitor
DN70798_c0_g2	1.08 × 10^1^	3.29 × 10^−23^	223.26	242.80	304.90	0.00	0.16	0.25	zinc metalloproteinase
DN69207_c33_g1	−1.12 × 10^1^	1.88 × 10^−93^	2.90	2.95	1.39	4108.93	10,335.52	4487.69	vitellogenin
DN54794_c0_g1	−1.18 × 10^1^	1.82 × 10^−52^	0.07	0.27	0.14	797.99	1193.92	401.69	vitellogenin 1
DN66165_c2_g1	−1.28 × 10^1^	5.76 × 10^−50^	0.03	0.11	0.08	735.01	809.74	454.49	triacylglycerol lipase
DN59442_c0_g1	−1.42 × 10^1^	4.16 × 10^−36^	0.05	0.04	0.08	1755.04	1544.04	1398.17	*none*
DN73776_c0_g1	−8.62 × 10^0^	1.39 × 10^−34^	0.07	0.06	0.05	25.42	27.33	23.90	vitellogenin receptor
DN61697_c0_g1	−1.58 × 10^1^	3.10 × 10^−34^	0.00	0.01	0.00	469.19	414.59	238.52	lysosomal aspartic protease; cathepsin D
DN50160_c11_g2	−2.02 × 10^1^	1.87 × 10^−33^	0.00	0.00	0.00	2541.30	4509.76	5446.56	glycoside hydrolase family 1
DN73353_c1_g2	−8.37 × 10^0^	1.32 × 10^−32^	2.28	1.25	1.80	191.16	167.25	134.58	sphingomyelin phosphodiesterase
DN47397_c1_g1	−1.90 × 10^1^	8.41 × 10^−32^	0.00	0.00	0.00	1315.10	2893.93	391.33	*none*

## Supplementary Materials

The following are available online at https://www.mdpi.com/2673-6284/9/4/21/s1, Table S1: Expression levels, contrasts, ascribed functions, and primers for differentially expressed *A. vittatum* genes and their associated transcripts (XLSX), Table S2: Association list mapping original transcript identifiers to NCBI accessions (TSV), Figure S1: Glutathione S-transferase phylogeny (PDF), Figure S2: Carboxylesterase phylogeny (PDF), Figure S3: Cytochrome P450 monooxygenase phylogeny (PDF). A set of supplementary figure legends is provided in the Word file, “SuppFigLeg.Avittatum_tct.docx”.
