# Supplementary material for: Transcriptome Sequencing of the Striped Cucumber Beetle, Acalymma vittatum (F.), Reveals Numerous Sex-Specific Transcripts and Xenobiotic Detoxification Genes
Source: BioTech (Basel). 2020 Oct 27;9(4):21. doi: 10.3390/biotech9040021 (PMC9258315; doi:10.3390/biotech9040021)
Supplement: Supplementary file 1 [file biotech-09-00021-s001.zip › SuppFigLeg.Avittatum_tct.docx]

***Supplementary Materials for***

Transcriptome Sequencing of the Striped Cucumber Beetle, *Acalymma vittatum* (F.), Reveals Numerous Sex-Specific Transcripts and Xenobiotic Detoxification Genes

Michael E. Sparks ^1^, David R. Nelson ^2^, Ariela I. Haber ^1^, Donald C. Weber ^1^ and Robert L. Harrison ^1,^*

^1^ Invasive Insect Biocontrol and Behavior Laboratory, USDA-ARS, Beltsville, MD 20705, USA; michael.sparks2@usda.gov (M.E.S.); ariela.haber@usda.gov (A.I.H.); don.weber@usda.gov (D.C.W.)

^2^ Department of Microbiology, Immunology and Biochemistry, University of Tennessee Health Science Center, Memphis, TN 38163, USA; drnelson1@gmail.com (D.R.N.)

***** Correspondence: robert.l.harrison@usda.gov (R.L.H.); Tel.: +1-301-504-5249

Supplementary Figure Legends

**Figure S1.** Glutathione S-transferase (GST) phylogeny. Leaf node identifiers are prefixed by species of origin: ALB ~ *Anoplophora glabripennis* (Asian long-horned beetle), CPB ~ *Leptinotarsa decemlineata* (Colorado potato beetle), SCB ~ *Acalymma vittatum* (striped cucumber beetle), TCAST ~ *Tribolium castaneum* (red flour beetle), and WCR ~ *Diabrotica virgifera virgifera* (western corn rootworm). GST classes are connoted by branch and leaf coloring: blue ~ theta, green ~ microsomal, brown ~ sigma, grey ~ omega, purple ~ prostaglandin E synthase, crimson-shaded red ~ delta, strawberry-shaded red ~ epsilon, and black ~ not classified. Branch lengths are based on a JTT model of protein evolution, and the scale bar denotes estimated amino acid substitutions per site. Node support per 100 bootstrap replicates is indicated using an italic font.

**Figure S2.** Carboxylesterase (COE) phylogeny. Leaf node identifier prefixes, branch lengths, scale bar, and node support are as described for Figure S1. COE classes are encoded by branch and leaf coloring: royal blue ~ β-esterases, purple ~ neuroligins, brown ~ acetylcholinesterases, turquoise ~ neurotactin, and black ~ palmitoleoyl COE NOTUM.

**Figure S3.** Cytochrome P450 monooxygenase (CYP) phylogeny. Leaf node identifier prefixes, branch lengths, scale bar, and node support are as described for Figure S1. CYP clans are indicated by branch and leaf coloring: green ~ CYP2, brown ~ CYP3, royal blue ~ CYP4, and red ~ mito.
