# Supplementary material for: Transcriptome Sequencing of the Striped Cucumber Beetle, Acalymma vittatum (F.), Reveals Numerous Sex-Specific Transcripts and Xenobiotic Detoxification Genes
Source: BioTech (Basel). 2020 Oct 27;9(4):21. doi: 10.3390/biotech9040021 (PMC9258315; doi:10.3390/biotech9040021)

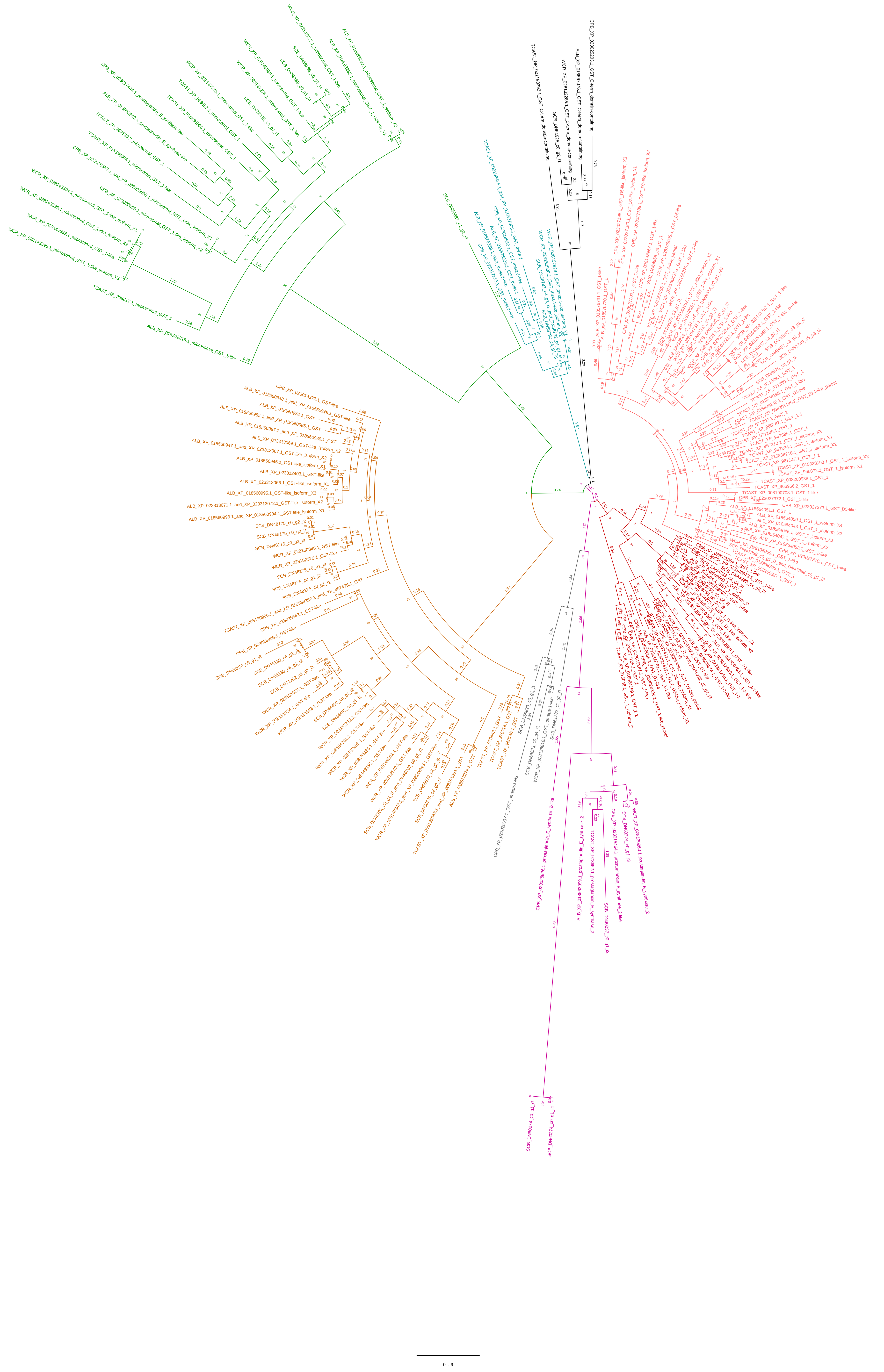

Supplement: Supplementary file 1 [file Figure_S1__GST_RAxML.pdf › Figure_S1__GST_RAxML.pdf]
